# Measuring Individual Differences in Generic Beliefs in Conspiracy Theories Across Cultures: Conspiracy Mentality Questionnaire

**DOI:** 10.3389/fpsyg.2013.00225

**Published:** 2013-04-30

**Authors:** Martin Bruder, Peter Haffke, Nick Neave, Nina Nouripanah, Roland Imhoff

**Affiliations:** ^1^Department of Psychology, Zukunftskolleg, University of KonstanzKonstanz, Germany; ^2^Department of Psychology, Northumbria UniversityNewcastle, UK; ^3^Department of Psychology, City University LondonLondon, UK; ^4^Department of Psychology, University of CologneCologne, Germany

**Keywords:** conspiracy theories, conspiracy mentality, generalized political attitudes, psychometric instrument, measurement equivalence, cross-cultural research

## Abstract

Conspiracy theories are ubiquitous when it comes to explaining political events and societal phenomena. Individuals differ not only in the degree to which they believe in specific conspiracy theories, but also in their general susceptibility to explanations based on such theories, that is, their *conspiracy mentality*. We present the Conspiracy Mentality Questionnaire (CMQ), an instrument designed to efficiently assess differences in the generic tendency to engage in conspiracist ideation within and across cultures. The CMQ is available in English, German, and Turkish. In four studies, we examined the CMQ’s factorial structure, reliability, measurement equivalence across cultures, and its convergent, discriminant, and predictive validity. Analyses based on a cross-cultural sample (Study 1a; *N* = 7,766) supported the conceptualization of conspiracy mentality as a one-dimensional construct across the three language versions of the CMQ that is stable across time (Study 1b; *N* = 141). Multi-group confirmatory factor analysis demonstrated cross-cultural measurement equivalence of the CMQ items. The instrument could therefore be used to examine differences in conspiracy mentality between European, North American, and Middle Eastern cultures. In Studies 2–4 (total *N* = 476), we report (re-)analyses of three datasets demonstrating the validity of the CMQ in student and working population samples in the UK and Germany. First, attesting to its convergent validity, the CMQ was highly correlated with another measure of generic conspiracy belief. Second, the CMQ showed patterns of meaningful associations with personality measures (e.g., Big Five dimensions, schizotypy), other generalized political attitudes (e.g., social dominance orientation and right-wing authoritarianism), and further individual differences (e.g., paranormal belief, lack of socio-political control). Finally, the CMQ predicted beliefs in specific conspiracy theories over and above other individual difference measures.

“Other centuries have only dabbled in conspiracy like amateurs. It is our (the Twentieth) century which has established conspiracy as a system of thought and a method of action” (Moscovici, 1987, p. 153).

## Introduction

Belief in conspiracy theories continues to thrive in the twenty-first century. In Western cultures, recent popular conspiracy theories have revolved – among other themes – around the perpetrators (and possible knowing bystanders) of the 9/11 attacks on the World Trade Center in New York (Stempel et al., 2007; Swami et al., 2010), the deaths of Princess Diana (Douglas and Sutton, 2008), and Osama bin Laden (Wood et al., 2012), and the scientific evidence for climate change (Leiserowitz, 2006). These new conspiracy theories take their place next to “classics” such as alleged plots concerning the assassination of John F. Kennedy (McHoskey, 1995), the cover-up of alien contact (Harrison and Thomas, 1997), or the origins of diseases such as HIV (Ross et al., 2006).

There is increasing evidence that there are stable individual differences in people’s tendency to believe in such conspiracy theories; if a person believes in one conspiracy theory, he or she will also be more likely to believe in other conspiracy theories (Swami et al., 2010). In fact, this tendency even extends to beliefs in mutually contradictory conspiracy theories, and to beliefs in fully fictitious conspiracy theories. Thus, those who believe that Princess Diana faked her own death are also more likely to believe that she was murdered (Wood et al., 2012); those who believe in “real-world conspiracy theories” (i.e., that John F. Kennedy fell victim to an organized conspiracy) are more likely to believe that there was a conspiracy behind the success of the Red Bull energy drink – a conspiracy theory that was purposely developed for a social psychology study (Swami et al., 2011).

This has led some researchers to propose that the endorsement of specific conspiracy theories depends to a large extent on individual differences in the general tendency to adopt such beliefs, that is, a general *conspiracy mentality* (Imhoff and Bruder, in press). This term was originally phrased by Moscovici (1987) who understood the notion of *conspiracy* as implying “that members of a confession, party, or ethnicity […] are united by an indissoluble secret bond. The object of such an alliance is to foment upheaval in society, pervert societal values, aggravate crises, promote defeat, and so on.” (p. 154). As such, a *conspiracy mentality* then describes the general propensity to subscribe to theories blaming a conspiracy of ill-intending individuals or groups for important societal phenomena or, in more abstract terms, the tendency to subscribe to “general conspiracist beliefs” (Swami et al., 2010). Usually, such theories contradict common explanations and allege that these events are caused by secret plots by groups of powerful individuals. Individual differences in conspiracy mentality have important consequences as they predict prejudice against powerful societal groups (Imhoff and Bruder, in press). Consequences of this may either be intentions to engage in political action designed to undermine the perceived conspiracy (Imhoff and Bruder, in press) or – if the conspiracy is perceived to be overpowering – political disengagement (Butler et al., 1995). Further, conspiracy beliefs are powerful predictors of critical health behaviors such as adherence to medication regimens (Bogart et al., 2010) and vaccination uptake (Kata, 2010).

There have been a number of initial efforts to measure individual differences in conspiracy mentality (sometimes called “conspiracist ideation”; Swami et al., 2011), the most prominent of which is Swami and colleagues’ Belief in Conspiracy Theories Inventory (BCTI; Swami et al., 2010, 2011). This consists of 15 items measuring beliefs in specific conspiracy theories (e.g., “A powerful and secretive group, known as the New World Order, are planning to eventually rule the world through an autonomous world government, which would replace sovereign governments.”) and has been used in the UK and continental Europe (Swami et al., 2011) as well as East Asia (Swami, 2012). The internal reliability of this scale has consistently been very good and it relates in meaningful ways to other individual difference variables such as: support for democratic principles, political cynicism, negative attitudes to authority, and low agreeableness (Swami et al., 2011).

However, scales measuring beliefs in specific conspiracy theories are closely bound to specific temporal and geographical contexts. In response to these limitations, it has been suggested that there is a need to assess the general tendency to believe in conspiracies in a way that is not dependent on the cultural familiarity of selected theories (Brotherton, French, and Pickering; in press) and also independent of knowledge about specific conspiracy theories which may vary between cultures. For example, it is unlikely that the BCTI item concerning the New World Order is equally familiar in all countries around the world. Hitherto, there have been two attempts to address this challenge, however, neither explicitly address the cross-cultural validity of the measurement instruments. First, Brotherton et al. (in press) have developed a 15-item Generic Conspiracist Beliefs Scale on the basis of an exploratory factor analysis of 75 items. They were able to differentiate between five major components of generic conspiracy beliefs: governmental conspiracies, extraterrestrial conspiracies, informational control conspiracies, personal well-being conspiracies, and malevolent global conspiracies. These dimensions are also reflected in the final scale. Second, Imhoff and Bruder (in press) developed a 12-item Conspiracy Mentality Scale. The items of this instrument not only avoid mentioning any specific alleged conspiracy, but also do not name any specific groups that may be responsible for a conspiracy (example item: “Most people do not recognize to what extent our life is determined by conspiracies that are concocted in secret”). Both the authors of the Generic Conspiracist Beliefs Scale and the Conspiracy Mentality Scale provide initial evidence for the convergent and discriminant validity of their instruments. However, neither scale has been validated in non-Western cultures and so far neither scale has been adopted by researchers other than the original authors.

We believe that these activities attest to the scientific relevance of developing valid, reliable, and efficient instruments to measure generic conspiracy beliefs. In this context, we will propose the Conspiracy Mentality Questionnaire (CMQ), a short (5-item) measure of generic conspiracy beliefs that we administered in a large (*N* = 7,766) international study spanning North American (US), Western European (UK and Ireland; Germany), and Middle Eastern (Turkey) cultures (see Study 1a). The cross-cultural dimension has so far been largely absent from research on conspiracy theories (for a notable exception examining the UK and Austria, see Swami et al., 2011). Developing that dimension would be highly desirable because it is likely that individual characteristics and cultural factors interact when it comes to the belief in specific conspiracy theories, and its consequences on attitudes and behaviors. In particular, a number of studies have shown that subcultures within national groups are differentially prone to belief in conspiracy theories. For example, African American and Latino communities in the US are particularly likely to endorse conspiracy theories claiming that HIV was spread to extinguish specific ethnic groups (Ross et al., 2006). Connecting these findings with research looking at individual differences in the propensity to believe in conspiracy theories is a promising endeavor – even more so when extending the perspective to different cultures around the globe. At the global level, conspiracy theories have been identified as a driving factor in the discourses of conflict in the Middle East (Pipes, 1998). Again, identifying how such broader societal phenomena relate to, and interact with, individual characteristics constitutes a worthwhile future research agenda. Our scale is designed to facilitate such future efforts.

### Overview and hypotheses

In Study 1a, we explore the factorial structure, assess the internal consistency, and test the measurement equivalence of the CMQ across its three language versions (English, German, Turkish). Study 1b tests the temporal stability of the CMQ across a 2-week interval. In Study 1a and three subsequent studies, we then examine the validity of the scale in predicting beliefs in 33 specific conspiracy theories. Further, in Studies 2–4, we test the convergent and discriminant validity of the construct of conspiracy mentality compared to other generalized political attitudes [right-wing authoritarianism (RWA), social dominance orientation (SDO)], personality measures (Big Five, schizotypy, paranoid ideation), and further individual differences measures (e.g., domains of paranormal belief, power-, and control-related self-perceptions, anomia, death anxiety).

On the basis of the pertinent literature we expect that conspiracy mentality will evince reliable correlations with many of these constructs. In particular, conspiracy mentality should be positively related to instruments gauging perceptions of low socio-political power (Abalakina-Paap et al., 1999) and anomia (Goertzel, 1994), paranoia and schizotypy (Grzesiak-Feldman and Ejsmont, 2008; Holm, 2009), paranormal belief (Ramsay, 2006), RWA (Adorno et al., 1950), and the personality dimensions of agreeableness (negatively) and openness to experience (positively) (Swami et al., 2010, 2011). At the same time, we expect that none of the correlations will reach a level that would cast doubt on the viability of conspiracy mentality as an independent construct. In addition, we hypothesize that conspiracy mentality will be the strongest and most consistent predictor of specific beliefs in conspiracy theories, even when controlling for other individual difference measures.

## Study 1a

Study 1a is the largest data collection to date measuring both beliefs in specific conspiracy theories and the general tendency to believe in conspiracy theories across four (groups of) countries: the US, UK, and Ireland, Germany, and Turkey. It allowed us (a) to examine the internal reliability and item loadings of the CMQ, (b) to test measurement equivalence of the instrument across three different language versions, (c) to investigate mean differences in conspiracy mentality across cultures as well as sex differences, and (d) to use the CMQ to predict the belief in specific conspiracy theories.

### Methods

#### Participants

Participants initially were recruited by solicitation e-mails to participants in prior online studies conducted by the first author and by posting the study on sites listing online studies (e.g., Reips and Lengler, 2005; Kathryn Gardner’s onlinepsychresearch.co.uk and John H. Krantz’ Psychological Research on the Net). The study proved highly popular and the link spread further through social networking sites, online groups and forums, and links in relevant articles of mainstream media outlets.

A total of 7,766 volunteers responded to 38 items measuring participants’ belief in conspiracy theories. 1.2% (*n* = 93) of the submissions were excluded due to (a) repeated submissions from one computer, (b) identical responses to all items, (c) self-reported *poor* command of the questionnaire language, or (d) missing responses to more than one questionnaire item. Further, (e) we only included participants with valid data on the five items measuring participants’ conspiracy mentality (see below). This resulted in a final sample of *N* = 7,673 participants (4,919 men, 2,694 women, 60 unreported) between the age of 18 and 67 years (*M* = 29.1, *SD* = 10.2). Participants’ were resident in Germany (*n* = 5,018), the US (*n* = 1,126), Turkey (*n* = 981), or the UK and Ireland (*n* = 548).

#### Materials and procedure

When arriving on the study website, participants could choose between one of three language versions (English, German, or Turkish). They then received a very broad and non-judgmental definition of the term *conspiracy theory*:

“A conspiracy theory is a theory that provides an alternative explanation to the established understanding of a historical or current event. Often, it is claimed that this event is the result of conscious manipulations by individuals or secretive powers. Due to our incomplete knowledge about the world, it can usually not ultimately be decided which explanatory model is true – the established understanding of an event or the respective conspiracy theory.”

We further informed participants that we were interested in their personal beliefs in such theories. Participants then provided demographic information and proceeded to the main questionnaire page starting with the following instructions: “For each of the statements below, please use the respective rating scale to indicate how likely it is in your opinion that the statement is true. Remember that there are no “objectively” right or wrong answers and that we are interested in your personal opinion.”

The 38 items administered in this study [dubbed the Conspiracy Theory Questionnaire (CTQ) by Darwin et al., 2011] contained 33 items measuring the belief in specific conspiracy theories and five items assessing participants’ general tendency to believe in conspiracies or their conspiracy mentality. We refer to these latter five items as the CMQ (see Appendix for complete item wording).

The items included in the study were either adapted from existing *ad hoc* scales assessing beliefs in conspiracy theories (e.g., Goertzel, 1994; Abalakina-Paap et al., 1999; Sjörberg, 2002) or developed drawing on the content of websites and online forums dedicated to conspiracy theories. Items assessing the belief in specific conspiracy theories covered, for example, conspiracies concerning assassinations (e.g., “Princess Diana’s death was an assassination rather than an accident.”), alien landings (e.g., “There are specialized government services who attempt to harass UFO witnesses into silence.”), technological developments (e.g., “A cure for most forms of cancer has already been found, but medical circles prefer to continue to extract research funding from governments and keep their findings secret.”), and secret activities of powerful organizations (e.g., “The Vatican Bank in Rome has close links to the Mafia.”). The five items assessing participants’ conspiracy mentality consisted of general statements capturing a conspirational view of world events.

Participants indicated on 11-point scales how likely they thought each item to be true from 0 (0% – *certainly not*) to 10 (100% – *certain*). Each scale point was additionally labeled with increasing probabilities in steps of 10 percentage points.

The original English items were translated into German and Turkish in a procedure similar to that proposed by Brislin (1970): Each item was first translated by a native speaker of the respective language and then translated back into English by a different person competent in both languages. Disagreements between the original and the backtranslated version and their implications for the translated version were resolved by discussion. In total, *n* = 5,026 participants completed the German version, *n* = 1,640 the English version, and *n* = 1,007 the Turkish version of the questionnaire.

After completing the questionnaire, participants had the opportunity to compare their own score with that of a normative sample, were debriefed, and thanked for their support.

#### Statistical analyses

Rather than being taken for granted, tests should be employed to determine whether a different language version of one questionnaire measures the same construct in an equivalent manner across different cultures (see Geisinger, 1994; van de Vijver and Hambleton, 1996; Hambleton, 2005). As a first step, we explored the factorial structure of the five items across the full sample and within each language version using principal component analyses.

Second, we tested for *measurement equivalence* (ME; also *measurement invariance* or *metric invariance*) across the three language versions. The focus here was on the invariant operation of the items and, in particular, on equivalent factor loadings across groups (Byrne, 2008, 2010). Using SPSS AMOS (Version 20), we conducted a multi-group confirmatory factor analysis and compared a configural model without constraints with a second model, in which factor loadings were constrained to be equal across groups. The second model is computed by freely estimating parameters for the first group and then constraining factor loadings for the other groups to be equal to those of the first one. Measurement equivalence can therefore be assumed if, through comparison, the models are deemed to be equally fitting of the data as this implies that the additional constraints did not substantially reduce model quality. Statistical testing for measurement equivalence is sometimes based on the comparison of each model’s χ^2^ values; however, this is a highly conservative method and is often seen as “impractical and unrealistic”(Byrne, 2009). When, as is the case in our analyses, the sample size is large, this often leads to statistically significant χ^2^ differences, even in case of negligible differences between the two models. We thus follow recommendations in the relevant literature and also compare changes in other fit indices between the unconstrained and the constrained model as a second source of information to evaluate measurement equivalence. In particular, we examined the comparative fit index (CFI), the Tucker–Lewis-Index (TLI), and the root mean square error of approximation (RMSEA). According to Hu and Bentler (1999), a cut-off value of 0.95 for CFI and TLI, and a value of less than 0.06 for RMSEA indicate a good model fit. For the RMSEA, values of up to 0.08 are still considered to indicate reasonable model fit (Browne and Cudeck, 1992) and values between 0.08 and 0.10 are considered to reveal mediocre fit (MacCallum et al., 1996). PCLOSE provides a one-sided test of the null hypothesis that RMSEA equals 0.05. PCLOSE-values larger than 0.05 indicate that the null hypothesis is not rejected – the model is then described as a “close fitting” model (Kenny, 2012). For changes in CFI and TLI values (ΔCFI, ΔTLI) in the context of examining measurement or structural equivalence (SE), Cheung and Rensvold (2002) proposed that only differences larger than 0.01 should lead one to reject the assumption of measurement equivalence. For ΔRMSEA, Chen (2007) recommended that only a difference of > 0.015 should lead to a rejection of measurement equivalence.

Testing the cross-cultural measurement properties of a psychometric scale can be extended to tests of SE. Here, the focus is on the unobserved (or latent) variables. Tests of SE can be used to check whether the factorial structure of a measurement instrument is the same across groups (Byrne, 2009).

### Results

#### Descriptive statistics

Item statistics (mean values, standard deviations, and item discrimination coefficients) for each of the five CMQ items for each of the language versions are presented in Table 1. Each of the three language versions had good (German: α = 0.84; English: α = 0.84) or acceptable (Turkish: α = 0.72) internal consistency and very good to medium item discrimination statistics (*r*_itc_ > 0.37).

**Table 1:** **Conspiracy Mentality Questionnaire item statistics for each of the three language versions**.

No.	Item	German version			English version			Turkish version		
		*M*	SD	*r_itc_*	*M*	SD	*r_itc_*	*M*	SD	*r_itc_*
1	I think that many very important things happen in the world, which the public is never informed about	8.04	2.37	0.63	8.00	2.14	0.62	8.84	1.53	0.48
2	I think that politicians usually do not tell us the true motives for their decisions	7.48	2.32	0.55	8.12	1.85	0.56	8.68	1.48	0.46
3	I think that government agencies closely monitor all citizens	3.35	2.94	0.60	3.90	2.93	0.62	3.72	2.72	0.37
4	I think that events which superficially seem to lack a connection are often the result of secret activities	4.59	2.93	0.72	4.69	2.72	0.70	6.97	2.28	0.65
5	I think that there are secret organizations that greatly influence political decisions	6.22	3.09	0.73	6.54	2.80	0.74	8.23	1.89	0.57

**r_itc_* = Corrected-Item-Scale-Correlation*.

**n*_German_ = 5,026, *n*_Engllish_ = 1,640, *n*_Turkish_ = 1,007*.

#### Factorial structure and model fit

**Figure 1:**
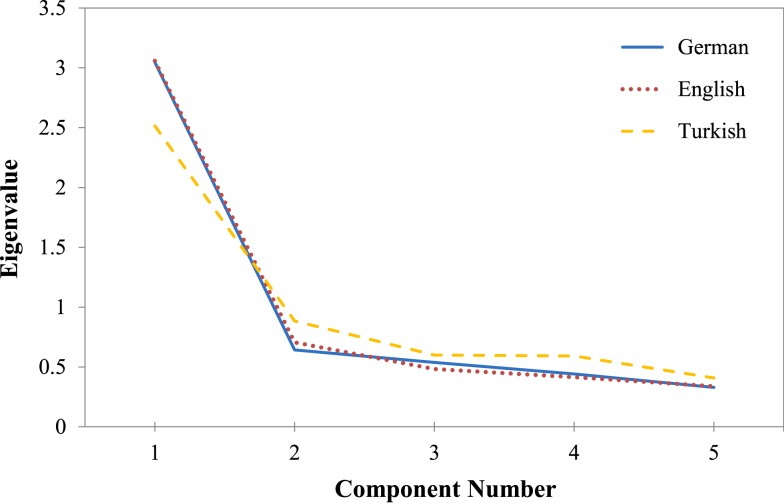
**Screen plot for exploratory factor analysis for each of the three language versions (Study 1a)**.

We first conducted an exploratory factor analysis across all participants to examine the factorial structure of the CMQ. The screen plot criterion suggested a one-factor solution explaining 60.6% of the variance. All factor loadings were larger than 0.71. Repeating this analysis for each language version separately also suggested a one-factor solution for each language version (see Figure 1). However, the proportion of explained variance was lower for the Turkish-speaking sample (50.3%) than for the German-speaking (61.0%) and English-speaking (61.2%) samples. Except for one item, all items strongly loaded on the extracted component (individual factor loadings were larger than 0.69 across all language versions). The one exception was the item “I think government agencies closely monitor all citizens” in the Turkish CMQ version with a still acceptable loading of 0.54.

#### Measurement equivalence across language versions

Using multi-group confirmatory factor analysis, we proceeded to test a one-factor model across the three different language versions. In this configural model, there were no constraints imposed on the parameters in the model; however, there was the minimal requirement that the number of factors and the number of items associated with each factor are equal across groups. Thus, this model tests whether the one-factor structure observed in the exploratory analyses can be confirmed when taking all three groups into account simultaneously.

As expected for our large sample, χ^2^ values were significant. However, examining alternative fit indices revealed very good model fit (see Table 2) with the CFI and the TLI being larger than 0.95 and the RMSEA smaller than 0.05. PCLOSE indicated a close fitting model. Thus we proceeded to tests of more restrictive models.

**Table 2:** **Tests for measurement and structural equivalence of the conspiracy mentality questionnaire across the three language versions (English, German, and Turkish)**.

	χ^2^	*df*	Δχ^2^	Δ*df*	CFI	ΔCFI	TLI	ΔTLI	RMSEA	PCLOSE	ΔRMSEA
No constraints imposed	288.07***	15	**–**	**–**	0.980	**–**	0.960	**–**	0.049	0.655	**–**
Measurement equivalence	347.01***	23	58.94***	8	0.976	0.004	0.969	−0.009	0.043	0.998	0.006
Structural equivalence	603.07***	25	256.06***	2	0.958	0.018	0.949	0.020	0.055	0.016	−0.012

CFI = comparative fit index; TLI = Tucker-Lewis-Index; RMSEA = root mean square error of approximation. ***p < 0.001.

In the measurement equivalence model, factor loadings were constraint to be equal across groups. Apart from the significant χ^2^ value, this model showed very good model fit which was highly similar to that of the unconstrained model. Again, the CFI, TLI, and RMSEA were within a value range that is generally accepted to indicate very good fit (see Table 2) and PCLOSE remained non-significant. In addition, the changes in all of these values between the configural model and the measurement equivalence model were minimal, all within a range supporting ME (Cheung and Rensvold, 2002; Chen, 2007). Thus, the CMQ is of equivalent measurement quality across all of its three language versions.

Building on the confirmatory results concerning measurement equivalence, we proceeded to test the SE of the CMQ across the three language versions. This is done by constraining both factor loadings and factor (co) variances to be equal across groups. The SE model showed acceptable model fit with both the CFI and the RMSEA falling within the range of well-fitting models and the TLI only falling very slightly short of the value of 0.95 (Hu and Bentler, 1999). Comparing the SE model with the less restrictive ME model revealed acceptable invariance only when using differences in RMSEA to assess model equivalence. These differences were minor (ΔRMSEA = −0.012) and within the suggested range (Chen, 2007). However, both changes in the CFI and the TLI are somewhat larger than would be expected for equivalent models (ΔCFI = 0.018; ΔTLI = 0.020). Inspection of the model modification indices showed that removing the constraint on the latent factor variance for the Turkish group to be equal to that of the two other groups would have resulted in a better fitting model. Indeed, such a model was highly similar to the measurement equivalence model with ΔCFI = 0.001, ΔTLI < 0.001, and ΔRMSEA < 0.001. Thus, although the measurement properties of the individual items of the CMQ are stable across language versions, questions remain as to whether the latent construct of conspiracy mentality is as coherent in Turkey as in Western European and North American countries.

#### Differences in mean levels of conspiracy beliefs

Having established that our five item measure operates well in all three language versions, this allowed us to investigate between-country differences in conspiracy mentality. We used a one-way ANOVA with Tukey HSD *post hoc* tests to examine mean differences between countries and independent groups *t*-tests to test sex differences between men and women in each country (Bonferroni-corrected α = 0.0125). Previous research in different disciplines suggests that conspiracy beliefs may be particularly rife in the Near East (e.g., Zonis and Joseph, 1994; Pipes, 1998; Gray, 2010) and we thus hypothesized that our Turkish sample would show higher mean scores than the samples from Western Europe and the US. As far as sex is concerned, Darwin et al. (2011) reported no significant differences. However, our larger sample allowed us to re-examine the idea that women may be more prone to believe in conspiracy theories than men. This may be because women are structurally disadvantaged in many societies and powerless individuals and groups are more susceptible to conspiracy beliefs (Abalakina-Paap et al., 1999). Furthermore, women are more likely to believe in paranormal phenomena (Irwin, 1993), a tendency that in itself is linked to higher conspiracy beliefs (Darwin et al., 2011).

Regarding differences in conspiracy mentality across the four countries, the ANOVA showed a significant main effect, *F*(3, 7669) = 120.32, *p* < 0.001, $\eta_{p}^{2}$ = 0.045. Tukey HSD follow-up comparisons revealed significant group differences between each pair of countries, *p*s < 0.01, except for the comparison between the US (*M* = 6.3; *SD* = 2.0) and UK/Ireland (*M* = 6.3; *SD* = 1.9), *p* = 1.00. Turkish participants’ (*M* = 7.3; *SD* = 2.1) conspiracy mentality was markedly higher than that of all other groups (*d*s > 0.58) whereas German participants scored lower than the other groups (*M* = 5.9; *SD* = 2.1). However, effect sizes of mean differences between Germany, the US, and the UK were small, |*d*s| < 0.20.

As far as sex differences are concerned, the only significant effect was observed for the US, *t*(1119) = 3.40, *p* = 0.001, *d* = 0.20, with women (*M* = 6.5; *SD* = 1.9) scoring slightly higher than men (*M* = 6.1; *SD* = 2.1).

In sum, the mean comparisons supported our contention that the susceptibility to believe in conspiracy theories is more pronounced in Near Eastern countries (i.e., Turkey) than in Western countries (Germany, UK/Ireland, US). In line with Darwin et al.’s (2011) findings, we also did not observe strong support for systematic sex differences, although the findings revealed that the hypothesis of women’s greater tendency to belief in conspiracy theories held in our US sample (despite a small effect size).

#### Predicting specific conspiracy beliefs

We also tested the extent to which conspiracy mentality as measured by the CMQ predicts endorsement of each of the 33 specific conspiracy theory items also included in the questionnaire. The CMQ correlated strongly with each of the specific conspiracy items, with correlations ranging from 0.37 to 0.76, each significant at a level of *p* < 0.001. The average correlation coefficient was $\bar{r}=0.58$, obtained by averaging Fisher’s *z* transformed correlation coefficients (StatSoft Inc., 2012).

## Study 1b

Study 1b examined the 2-week test-retest reliability of the CMQ.

### Method

The German version of the CMQ was administered twice at the end of an otherwise unrelated study. The time lag between the invitations to the two assessments was 15 days. All participants were members of a German university. Of 177 participants who completed the CMQ at t1, 75% or 133 participants (82 women, 50 men, 1 unidentified; *M*_Age_ = 24.27, *SD*_Age_ = 5.21) also completed assessment at t2. Dropout was independent of CMQ scores at t1, *t*(175) = 1.07, *p* = 0.28.

### Results

Internal consistency of the 5-item CMQ was satisfactory both at t1 (α = 0.77) and t2 (α = 0.82). The correlation between the two assessment points was 0.84.

## Summary Studies 1a and 1b

Study 1a established the factorial structure and internal consistency of a new 5-item psychometric instrument – the CMQ – designed to measure the general tendency to believe in conspiracy theories that characterizes a conspiracy mentality. It further provided support for measurement equivalence of three different language versions of the CMQ (English, German, and Turkish). As far as SE was concerned, there remained some doubts as to whether a one-factorial structure can adequately capture the construct of a “conspiracy mentality” in Turkey. Comparisons of mean levels of conspiracy mentality revealed higher levels in Turkey than in Western European and North American countries. Sex differences were small with the only significant sex effect showing higher levels of conspiracy mentality among US women than men. Study 1b demonstrated satisfactory test-retest reliability of the CMQ over a 2-week interval.

## Study 2

Studies 2–4 are concerned with the convergent and discriminant validity of the CMQ with other individual differences measures. Study 2, a focused re-analysis of data presented by Darwin et al. (2011) examined the relations between conspiracy mentality as assessed by the CMQ and (a) people’s tendency to belief in paranormal events, (b) paranoid ideation, and (c) a schizotypal personality disposition. A tendency to believe in the paranormal manifests itself in the acceptance of events and processes that accepted science currently deems impossible; paranoid ideation is characterized by fears and suspicions about physical and psychological threats potentially posed by social agents; and schizotypy is a mild form of schizophrenia that involves suspicion, magical thinking, paranoid beliefs, and different forms of social anxiety. All three of these constructs have been theoretically and empirically linked to beliefs in conspiracy theories (Ramsay, 2006; Grzesiak-Feldman and Ejsmont, 2008; Holm, 2009; Darwin et al., 2011).

### Method

#### Participants

The sample consisted of 120 students (60 men and 60 women) of a university in the UK. All participants were between 18 and 30 years of age. One female participant was excluded because of missing values needed for the computation of the CMQ score.

#### Materials and procedure

After obtaining participants’ informed consent, four questionnaires were presented in random order. First, the study contained the same 38 items as Study 1a assessing (a) participants’ conspiracy mentality (CMQ; α = 0.85) and (b) their beliefs in specific conspiracy theories using the same 11-point response scales. Second, the Paranormal Belief Scale (PBS; Tobacyk and Milford, 1983) measures seven factors of paranormal belief (traditional religious belief, psi beliefs, witchcraft, superstition, spiritualism, extraordinary life forms, and precognition) using 25 items. Participants indicated the extent to which they agreed with each statement on a 5-point scale (1 = *strongly disagree*, 5 = *strongly agree*). Third, the Paranoid Ideation Scale (PIS; Fenigstein and Vanable, 1992) consists of 20 statements and measures paranoid experiences and beliefs. Participants indicated the extent to which they agreed with each item on a 5-point scale (1 = *not at all applicable to me*, 5 = *extremely applicable to me*). Fourth, the Schizotypal Personality Questionnaire (SPQ; Raine and Benishay, 1995) consists of 22 items with three subscales: cognitive-perceptual deficits (eight items), interpersonal deficit (eight items), and disorganization (six items). The SPQ measures the DSM-defined schizotypal personality disorder. Items are answered using a *yes* vs. *no* response format.

### Results

#### Correlations with individual difference measures

Following the original approach by Darwin et al. (2011), we computed partial correlation coefficients (controlling for participants’ sex) between participants’ self-reported conspiracy mentality and their tendency to engage in paranoid ideation, to hold paranormal beliefs, and their schizotypal personality disposition (see Table 3). Conspiracy mentality was strongly (0.40 < *r* < 0.50) associated with paranoid ideation and the total score of the PBS (mainly driven by a strong correlation with psi beliefs). There were also significant medium-sized correlations with all SPQ subscales. The superstition and precognition subscales of the PBS were not significantly correlated with the CMQ. No correlation of any subscale with the CMQ was larger than *r*_p_ = 0.50.

**Table 3:** **Intercorrelations between individual difference measures (Study 2)**.

		1	2	3	4	5	6	7	8	9	10	11	12	13	14
1	CMQ	–													
2	PIS	0.449***	–												
3	PBS_RB_	0.252**	0.289**	–											
4	PBS_PSI_	0.481***	0.376***	0.467***	–										
5	PBS_WC_	0.387***	0.288**	0.346***	0.666***	–									
6	PBS_SP_	0.420***	0.387***	0.564***	0.662***	0.664***	–								
7	PBS_SU_	0.170	0.311***	0.303***	0.328***	0.348***	0.417***	–							
8	PBS_ELF_	0.353***	0.245**	0.460***	0.407***	0.396***	0.487***	0.255**	–						
9	PBS_PC_	0.212*	0.290**	0.407***	0.452***	0.394***	0.628***	0.394***	0.203**	–					
10	PBS_total_	0.456***	0.430***	0.720***	0.794***	0.766***	0.882***	0.569***	0.644***	0.667***	–				
11	SPQ_CPD_	0.353***	0.558***	0.489***	0.515***	0.470***	0.625***	0.369***	0.399***	0.435***	0.656***	–			
12	SPQ_ID_	0.177	0.437***	0.074	0.218*	0.319***	0.221*	0.263*	−0.039	0.172	0.238**	0.311***	–		
13	SPQ_DISS_	0.301***	0.465***	0.176	0.182*	0.173	0.184*	0.092	0.076	0.051	0.191*	0.360***	0.297**	–	
14	SPQ_total_	0.359***	0.649***	0.308***	0.399***	0.432***	0.446***	0.329***	0.170	0.289**	0.471***	0.717***	0.787***	0.707***	–

*Partial correlations controlling for participants’ sex. CMQ  = conspiracy mentality questionnaire, PIS  = paranoid ideation scale, PBS_RB_ = PBS subscale Religious beliefs, PBS_PSI_ = PBS subscale Psi beliefs, PBS_WC_ = PBS subscale Witchcraft, PBS_SP_ = PBS subscale Spiritualism, PBS_SU_ = PBS subscale Superstition, PBS_ELF_ = PBS subscale Extraordinary life forms, PBS_PC_ = PBS subscale Precognition, PBS_total_ = Paranormal Belief Scale total score, SPQ_CPD_ = Schizotypy subscale Cognitive- perceptual deficit, SPQ_ID_ = Schizotypy subscale Interpersonal deficit, SPQ_DISS_ = Schizotypy subscale Disorganization, SPQ_total_ = Schizotypy total score*.

*p < 0.05. **p < 0.01. ***p < 0.001.

#### Predicting specific conspiracy beliefs

We again tested the extent to which participants’ conspiracy mentality predicted each item measuring belief in a specific conspiracy theory. Replicating results of Study 1a, all specific conspiracy beliefs were significantly predicted by the CMQ with correlations ranging from 0.30 to 0.81 (all *p*s < 0.001). The average correlation coefficient was $\bar{r}=0.55$.

As a next step, we tested whether the association between conspiracy mentality and specific conspiracy beliefs remained stable when controlling for paranormal belief (with its seven subscales), paranoid ideation, and schizotypy (three subscales). Because all 12 predictors (including the CMQ) were substantially correlated, we used a stepwise regression procedure to avoid problems of multicollinearity. Each time a new variable was entered into the model (inclusion criterion *p* < 0.05), the significance of already entered variables was re-examined. If any *p*-values then exceeded the exclusion criterion of *p* > 0.10, the predictor with the highest *p*-value was removed before refitting the model. This procedure was repeated until no further variables met the inclusion or exclusion criterion. In 30 of 33 regressions, the CMQ explained the largest part of the variance (0.29 < β < 0.81; average β = 0.53). The remaining three specific conspiracy beliefs included two items concerning alleged cover-ups of alien contact. For both these items, the psi beliefs subscale of the PBS was the strongest predictor (βs = 0.38 and 0.35) followed by the CMQ (βs = 0.32 and 0.30) and no other significant predictors. Finally, the allegation that the US Apollo program never landed on the moon was again most strongly predicted by psi beliefs (β = 0.24) as well as the religious belief subscale of the PBS (β = 0.21). The CMQ remained a marginally significant predictor with β = 0.18, *p* = 0.050. Apart from the psi beliefs and superstition subscales of the PBS, which significantly predicted belief in five specific conspiracy theories, no other predictor significantly added explained variance to more than two specific conspiracy beliefs.

## Summary

Study 2 took a first step in placing conspiracy mentality as assessed by the CMQ within a wider nomological network of other relevant constructs. CMQ scores showed substantial correlations with scales measuring paranoid ideation and schizotypal personality. They were also associated with several aspects of paranormal belief. Although these correlations demonstrate that conspiracy mentality is related to other constructs in meaningful and predictable ways, none of the correlations was of a size that would raise doubts as to whether conspiracy mentality is viable as an independent construct. As in Study 1a, conspiracy mentality was a reliable predictor of beliefs in specific conspiracy theories, although the tendency to believe in psi phenomena also reliably predicted conspiracy beliefs concerning space flight and alien contact.

## Study 3

Study 2 recruited a sample of students from a British university. The strong reliance on young and highly educated student samples to develop psychometric scales and test psychological theories has been much discussed and criticized (Peterson, 2001; Henrich et al., 2010). Thus, in Study 3 we recruited a sample drawn from the British working population. In addition to replicating the internal structure of the CMQ and its association with paranoid ideation, Study 3 also aimed at confirming the association between conspiracy mentality and schizotypy using a different measure of a schizotypal personality disposition.

### Method

#### Participants

An opportunity sample of 76 full-time employes (mainly in the insurance and financial industry; no students) participated in the study. One male participant had to be excluded because of missing data on the 5-item CMQ. The final sample consisted of 28 men and 47 women between the age of 22 and 54 years (*M* = 31.3; *SD* = 8.1).

#### Materials and procedure

Participants completed the following self-report scales: First, they responded to the same 38 items assessing conspiracy beliefs as in the previous two studies. The 5-item CMQ again proved reliable (α = 0.73). They then indicated their agreement (*yes* vs. *no*) with the items of two subscales of the Oxford-Liverpool Inventory of Feelings and Experiences (O-LIFE; Mason et al., 1995). The 30 items of the “Unusual Experiences” subscale refer to positive symptoms of a psychosis (e.g., hallucinatory and magical thinking) whereas the 23 items of the “Cognitive Disorganization” subscale describe difficulties in the areas of attention, concentration, and decision-making, as well as a sense of purposelessness, moodiness, and social anxiety. Finally, they completed the Paranoid Ideation Scale (PIS; Fenigstein and Vanable, 1992) already used in Study 2.

### Results

#### Correlations with individual difference measures

We calculated correlation coefficients between participants’ conspiracy mentality as measured by the CMQ and their tendency to make unusual experiences (*r* = 0.53), to be cognitively disorganized (*r* = 0.42), and to engage in paranoid ideation (*r* = 0.50; all *p*s < 0.001).

#### Predicting specific conspiracy beliefs

As in the two previous studies, we computed zero-order correlation coefficients between participants’ conspiracy mentality and their belief in 33 specific conspiracy theories. We again tested the extent to which participants’ conspiracy mentality predicted each of the items measuring the belief in a specific conspiracy theory. Again, all specific conspiracy beliefs were significantly predicted by conspiracy mentality (all *p*s < 0.05) with correlations ranging from 0.20 to 0.69. The average correlation coefficient was $\bar{r}=0.50$.

As in Study 2, we conducted 33 stepwise regression analyses to examine the predictive validity of conspiracy mentality when controlling for other individual differences measures. In 28 of 33 regressions, the CMQ was the strongest predictor for the belief in specific conspiracy theories (0.26 < β < 0.69; average β = 0.47), followed by the PIS (predicting five conspiracy beliefs with βs between 0.21 and 0.30) and the O-LIFE subscale Cognitive Disorganization (predicting four conspiracy beliefs with βs between 0.21 and 0.28). For three specific conspiracy belief, PIS (βs = 44. and 0.41) and Cognitive Disorganization (β = 0.30) were the strongest predictors, followed by the CMQ (βs = 0.31, 0.27, and 0.29, respectively). The former two conspiracy beliefs are concerned with the complexity of terror networks and the government’s ability to uncover individual choices in parliamentary elections. The latter belief is concerned with the withholding of cures for cancer to further extract research funding. For the item stating that better car engines have already been developed but are not being made commercially available, the O-LIFE subscale Unusual Experiences was the only significant predictor (β = 0.27, *p* = 0.02). Finally, the item alleging the deliberate spread of HIV among minorities was not significantly related to any of the predictors (*p*s > 0.26).

## Summary

Study 3 replicated the findings of Study 2 with respect to the association between conspiracy mentality and paranoid ideation and schizotypy in a non-student sample. Again, as expected, conspiracy mentality was found to be substantially related to both of these constructs, but not identical to them. As before, conspiracy mentality did well in predicting beliefs in specific conspiracy theories.

## Study 4

In Study 4 we reanalyzed data presented by Imhoff and Bruder (in press) to address four main goals: First, we wanted to test the convergent validity of the CMQ with another validated measure of generic conspiracy beliefs. Second, we wanted to examine its discriminant validity with a broader set of more fundamental personality characteristics and generalized (political) attitudes. Third, we wanted to replicate the predictive validity of the CMQ for specific conspiracy beliefs in another national and language context (Germany instead of the UK). Fourth, we wanted to provide a more severe test of the CMQ’s predictive validity by controlling for a large set of individual difference measures when predicting specific conspiracy beliefs.

### Method

#### Participants

The original data collected by Imhoff and Bruder (in press) consisted of 280 participants, recruited via an e-mail list of a German university. Six participants were excluded due to missing values on the 5-item CMQ (α = 0.78). The data subjected to re-analysis therefore comprised 96 male and 178 female participants aged 16 to 69 years (*M* = 25.6; *SD* = 8.1).

#### Materials and procedure

Participants in this study first rated 32 social groups on their levels of perceived power, likeability, and realistic and symbolic threat. For detailed analyses of these ratings, see Imhoff and Bruder (in press).

Participants then completed the 5-item CMQ as well as the same 33 specific conspiracy belief items as were used in Studies 1–3. In addition, they responded to the following individual difference measures:

1. **Conspiracy Mentality Scale**: This is a 12-item measure (α = 0.89) of generic conspiracy beliefs. Items were either adapted from the existing literature (Adorno et al., 1950) or purpose-designed for the study. Items (e.g., “Those at the top do whatever they want”) were rated on 7-point scales ranging from *do not agree* to *fully agree*.
2. **Right-wing authoritarianism** was measured using Funke’s (2005) 12-item scale covering conventionalism, authoritarian aggression, and authoritarian submission (α = 0.81; example item: “The withdrawal from tradition will turn out to be a fatal fault one day.”).
3. **Social dominance orientation** was assessed with the German adaptation (von Collani, 2002) of the original 16-item *Social Dominance Orientation* scale (Pratto et al., 1994; α = 0.89; example item: “To get ahead in life, it is sometimes necessary to step on other groups.”).
4. **Perceptions of control** in the personal (α = 0.61), interpersonal (α = 0.79), and socio-political (α = 0.70) domains was measured using Paulhus (1983) *Spheres of Control* scale. Example item: “Even when I’m feeling self-confident about most things, I still seem to lack the ability to control social situations” (interpersonal control).
5. **Perceptions of powerlessness** were measured using a scale consisting of seven items that were partly taken from the literature and partly purpose-designed (α = 0.70; e.g., “The problems of life are sometimes too big for me”).
6. **Anomia** is a concept that describes the perception that the complexity of modern societies has become unintelligible. The construct was measured using a 7-item scale (α = 0.74). Example item: “Things have gotten so confusing that nobody really knows what is what anymore.”
7. **Death anxiety** was assessed using the five highest-loading items (items 2, 3, 12, 14, 18) of the first factor of the *Revised Death Anxiety Scale* (RDAS; Thorson and Powell, 1992; α = 0.92; example item: “The idea of never thinking again after I die frightens me”).
8. **Anthropomorphism**, the tendency to attribute human-like characteristics such as agency to inanimate objects (e.g., “To what extent does a television set experience emotions?), was measured using the 15-item *Individual Differences in Anthropomorphism Questionnaire* (IDAQ; Waytz et al., 2010; α = 0.89).
9. **Big Five personality** dimensions were assessed using the Big Five inventory (BFI-K; Rammstedt and John, 2005; example item: “I am rather reserved, shy”). This scale consists of 21 items assessing extraversion (α = 0.85), agreeableness (α = 0.59), conscientiousness (α = 0.69), neuroticism (α = 0.79), and openness to experience (α = 0.73).

### Results

We computed partial correlations (controlling for participant sex) between the CMQ and every other individual difference measure (see Table 4). Attesting to the convergent validity of the CMQ, it was very highly correlated with the Conspiracy Mentality Scale (*r* = 0.82, *p* < 0.001). A medium-size correlation also emerged with anthropomorphism (*r* = 0.36, *p* < 0.001) and RWA (*r* = 0.28, *p* < 0.001). Small-to-medium correlations (all |*r*s| < 0.25) also existed with powerlessness, anomia, social dominance orientation, perceived socio-political control, and agreeableness. As could be expected, the former three of these were positively related to conspiracy mentality, whereas the latter two were negatively related to conspiracy mentality. The remaining measures were not reliably associated with the CMQ.

**Table 4:** **Intercorrelations between individual difference measures (Study 4)**.

		1	2	3	4	5	6	7	8	9	10	11	12	13	14	15	16
1	CMQ	–															
2	CM	0.817***	–														
3	RWA	0.276***	0.287***	–													
4	SDO	0.156*	0.125*	0.522***	–												
5	B5_E_	0.029	−0.028	−0.048	−0.052	–											
6	B5_A_	−0.141*	−0.099	−0.143	−0.238***	0.093	–										
7	B5_C_	0.008	0.009	0.111	0.024	0.122*	−0.045	–									
8	B5_N_	0.057	0.068	0.026	0.006	−0.298***	−0.048	−0.226***	–								
9	B5_O_	0.058	0.073	−0.149*	−0.129*	0.126*	0.063	0.134*	0.082	–							
10	AN	0.356***	0.306***	0.203***	0.087	0.018	−0.089	−0.005	0.026	0.053	–						
11	DA	0.103	0.078	0.055	0.055	−0.026	−0.071	−0.075	0.222***	−0.041	0.117	–					
12	A	0.224***	0.269***	0.205***	0.146*	−0.410***	−0.129	−0.366***	0.550***	−0.146*	0.144*	0.143*	–				
13	PL	0.230***	0.269***	0.155*	0.067	−0.294***	0.010	−0.332***	0.486***	−0.090	0.155*	0.130*	0.723***	–			
14	PC_P_	0.029	0.039	0.087	0.030	0.141*	−0.055	0.544***	−0.226***	0.112	−0.013	−0.045	−0.364***	−0.380***	–		
15	PC_IP_	−0.091	−0.081	−0.090	−0.061	0.621***	0.113	0.344***	−0.345***	0.213***	0.020	−0.070	−0.558***	−0.495***	0.395***	–	
16	PC_SP_	−0.220***	−0.218***	−0.289***	−0.277***	0.182**	0.095	0.091	−0.156*	0.222***	−0.034	−0.137*	−0.348***	−0.370***	0.112	0.280***	–

Partial correlations controlling for participants’ sex. CMQ = Conspiracy Mentality Questionnaire, CM = Conspiracy Mentality Scale, RWA = right-wing authoritarianism, SDO = social dominance orientation, B5_E_ = Big Five: Extraversion, B5_A_ = Big Five: Agreeableness, B5_C_ = Big Five: Conscientiousness, B5_N_ = Big Five: Neuroticism, B5_O_ = Big Five: Openness, AN = Anthropomorphism, DA = death anxiety, A = Anomia, PL = powerlessness, PC_P_ = Perceived control: personal efficacy, PC_IP_ = Perceived Control: interpersonal control, PC_SP_ = Perceived Control: socio-political control. *p < 0.05. **p < 0.01. ***p < 0.001.

#### Predicting specific conspiracy beliefs

We again examined the extent to which the CMQ predicted each of the 33 specific conspiracy beliefs and – in line with the previous findings – found medium-sized to strong correlations, ranging from 0.32 to 0.68 (all *p*s < 0.001). The average correlation coefficient was $\bar{r}=0.50$.

Once again, we also conducted 33 stepwise regression analyses regressing specific conspiracy beliefs simultaneously on the CMQ and all other individual difference measures included in the study (apart from the Conspiracy Mentality Scale). For all 33 items, the CMQ remained the most powerful predictor of specific conspiracy beliefs with βs ranging from 0.31 to 0.65 (average β = 0.46). Of the other individual difference measures, the most powerful predictors of specific conspiracy beliefs were (a) RWA (predicting 18 conspiracy beliefs with average β = 0.17) and (b) anthropomorphism (predicting nine conspiracy beliefs with average β = 0.16). The Big Five dimensions, SDO, and perceived personal, interpersonal, and socio-political control did not systematically predict specific conspiracy beliefs (all subscales were significant predictors of less than five beliefs with all |βs| < 0.19).

## Summary

Study 4 attested to the *convergent validity* of the CMQ. In particular, it was very highly correlated with the Conspiracy Mentality Scale, a measure that has been shown to meaningfully predict socio-political attitudes (Imhoff and Bruder, in press). Further indication of convergent validity came from substantial correlations with anthropomorphism and RWA. Anthropomorphism refers to the tendency to assume human-like tendencies in inanimate objects. Although not obvious at first sight, such a tendency might be related to conspirational thinking as both types of thinking styles assume agency where there may be none, like intentionality in a TV set or secret activities of conspirators in the absence of good evidence for such activities. As for RWA, the original conceptualization of RWA (Adorno et al., 1950) included a construct called *projectivity* which is closely related to our conceptualization of a conspiracy mentality. The relevant subscale of the California F-Scale of authoritarianism (Adorno et al., 1950) included items such as “Most people don’t realize how much our lives are controlled by plots hatched in secret places.”

Study 4 also demonstrated the *discriminant validity* of the CMQ in that all correlations with other individual difference measures were not at a level that shed doubt on the conceptualization of conspiracy mentality as an independent construct. In particular, notwithstanding the meaningful correlation with RWA, neither RWA nor SDO as the two major generalized political attitudes showed more than a medium-sized correlation with the CMQ. Also, the only (negative) association between the CMQ and the Big Five personality dimensions was observed for agreeableness (see Swami et al., 2011, for convergent findings); this correlation was reliable but only small in size.

The study also provided good evidence for the *predictive validity* of the CMQ: the instrument strongly predicted specific conspiracy beliefs even when controlling for other individual difference measures. The predictive power of the scale was upheld even in another national and language context.

## Discussion

The main purpose of our analyses was to examine the factorial structure and internal consistency, the measurement equivalence across cultures, and the validity of the 5-item CMQ – a psychometric instrument designed to assess individual differences in *conspiracy mentality*.

Regarding the factorial structure of the CMQ, our data were consistent with the assumption that conspiracy mentality – at its core – constitutes a one-dimensional construct. In each of the English, German, and Turkish language versions, exploratory factor analyses strongly suggested a one-factor solution with satisfactory loadings of all individual items; accordingly, internal consistency of the scale was adequate in all studies. This is consistent with the fact that longer instruments are able to identify subscales of generic conspiracy beliefs. Brotherton et al. (in press) Generic Conspiracist Beliefs Scale does just that by differentiating between conspiracy theories related to government malfeasance, extraterrestrial cover-up, control of information, etc. In order to successfully identify subscales, the instrument must contain a sufficient number of items with relatively specific information about possible perpetrators and topics of conspiracies without explicitly referring to any existing conspiracy theory. This approach comes with dangers. First, any specification of content-related aspects such as the topic of the conspiracy (e.g., “New and advanced technology which would harm current industry is being suppressed”; Brotherton et al., in press) renders cross-cultural comparisons more difficult. For example, whereas in some countries the transition from old to new technologies may simply be a matter of market forces, in other countries new technologies may actually be suppressed for political or economic ends. Although an item such as the one by Brotherton et al. (in press) above may be a good indicator of conspiracy mentality in the former type of country (e.g., democracies), answers to this item may reflect appropriate skepticism and low gullibility rather than conspiracy mentality in the latter type of country (e.g., autocracies). Second, any specification of content-related aspects makes a scale more susceptible to the influence of current political events (e.g., the Fukushima Daiichi disaster in 2011 may have temporarily or lastingly changed attitudes toward some new technologies in certain countries but not others). Thus, although there is doubtlessly substantial benefit in a fine-grained assessment of conspiracy beliefs, we suggest that there are also benefits in a short generic measure focusing on the central construct of conspiracy mentality. The two approaches should therefore be considered as complementary. As Brotherton et al. (in press) have themselves argued, for most purposes, it will be sufficient to establish an overall score measuring the tendency to engage in conspiracist ideation. The CMQ provides a highly economical instrument to do just that, for which measurement equivalence has been established across markedly different cultures. Brotherton et al. (in press) Generic Conspiracist Beliefs Scale, instead, may be better suited for exploring domain-specific differences in conspiracy beliefs. However, its measurement and SE have yet to be established before it can be used in non-English-speaking countries.

Our analyses concerning the measurement equivalence of the CMQ across its three language versions provided initial support for the idea that it is possible to assess conspiracy mentality across different cultures. In fact, constraining item loadings to be equal across language versions did not substantially reduce model fit. Thus, items “function” equally well in English-, German-, and Turkish-speaking contexts despite some questions as to whether the construct of conspiracy mentality is as well captured by a one-dimensional scale in a Turkish context than in the other two contexts. This finding illustrates the potential complementarity of our measure with other, more detailed and culture-dependent measures of conspiracy beliefs. Our scale helped to identify a need to examine the structure of conspiracy beliefs in the Middle East using more extensive measures such as the one developed by Brotherton et al. (in press). Study 1a also provided support for the utility of the CMQ in describing mean differences in conspiracy beliefs. At a cross-cultural level, Turkish participants were more prone to believe in conspiracy theories than participants from Western countries (Germany, UK/Ireland, US), who did not systematically differ in their conspiracy mentality. This is consistent with suggestions that conspiracy beliefs are particularly rife in the Middle East (e.g., Pipes, 1998). Within cultures, we observed that women in the US, but not in the other countries, scored higher on conspiracy mentality than did men. This is a powerful reminder that even research focusing on sex differences does well in examining men and women in more than one culture before claiming to have uncovered “essential” (rather than culture-specific) differences between the sexes.

Our studies provide ample evidence for the convergent, discriminant, and predictive validity of the CMQ. Study 4 showed that it is closely associated with the Conspiracy Mentality Scale (Imhoff and Bruder, in press), a measure that has been shown to meaningfully predict intergroup and political attitudes. Further evidence for the convergent validity of the scale comes from meaningful predicted correlations with other generalized political attitudes (RWA, social dominance orientation), personality measures (schizotypy, paranoid ideation, agreeableness), and further individual differences measures (e.g., most domains of paranormal belief, anomia, anthropomorphism, perceptions of powerlessness and lack of socio-political control). Beyond these associations, there exist a plethora of potentially meaningful and important relationships to other constructs. For example, recent articles discussed the influence of uncertainty and perceptions of morality (Van Prooijen and Jostmann, 2013) as well as the influence of anxiety-inducing situations (Grzesiak-Feldman, 2013) on conspiracist ideation. One may also want to explore whether people with strong imaginative abilities (i.e., high fantasy proneness or absorption) or those who are more susceptible to outside influence (e.g., those high in hypnotic suggestibility) are more likely to believe in conspiracy theories, or whether and in what way personality variables in general interact with conspiracy mentality over time. However, these questions will have to be addressed in future research. For the initial evaluation of the CMQ, we purposely focused on those constructs that have been mentioned in the literature on conspiracy beliefs and ascertained that the CMQ relates to these constructs in predictable ways.

The relationship between perceptions of control and conspiracy beliefs warrants some further discussion. Conspiracy beliefs have long been linked to low levels of societal power and control. For example, those threatened by unemployment as well as ethnic minorities are more likely to believe in conspiracy theories (Goertzel, 1994; Abalakina-Paap et al., 1999). Consistent with these findings, we also observed a negative association between conspiracy mentality and perceived socio-political control as well as a positive association between conspiracy mentality and anomia. However, in our data there was no relationship between conspiracy mentality and lack of personal or interpersonal control despite recent evidence that inducing perceptions of low personal control also increases the attribution of ambiguous events to conspiracies (Whitson and Galinsky, 2008; Kay et al., 2009). We argue that the difference lies in the functionality of conspiracy beliefs that differs between our correlational design and experimental designs such as Whitson and Galinsky’s (2008) due to differences in attribution. For experimentally induced control deprivation, beliefs in conspiracies constitute an opportunity to engage in compensatory action: Seeing the plot behind the curtains helps to regain a sense of control. We argue that the situation is different for self-reported low levels of personal control that are predominantly internally attributed (as measured, e.g., by the item “It’s pointless to keep working on something that’s too difficult for me” of Paulhus’, 1983, *Spheres of Control* scale). Here, adopting a belief about how others secretly try to gain control over the world would not constitute a functional way of regaining a sense of control, precisely because this lack of control is internally attributed. If own failures were externally attributed (e.g., if there was an item stating “Others confront me with much harder tasks than my competitors”), endorsing conspiracy beliefs may be functional in regaining feelings of control (“I am not incompetent, rather, ill-intending individuals are against me”). The same logic applies to the *Spheres of Control* subscale of interpersonal control that also targets internally attributed lack of control (e.g., “I’m not good at guiding the course of a conversation with several others”). However, this is markedly different for the subscale tapping into socio-political control which explicitly introduces attributions to external causes (e.g., “Bad economic conditions are caused by world events that are beyond our control”). Thus, it should be expected that conspiracy mentality as a stable generalized political attitude (Imhoff and Bruder, in press) is related to levels of socio-political control but not to momentary fluctuations in perceived personal or interpersonal control. Indeed this is what we observed.

Although conspiracy mentality relates to a large number of individual difference measures in meaningful ways, none of these correlations reaches a level that casts doubt on its viability as an independent construct. In particular, whereas the CMQ’s correlation with the Conspiracy Mentality Scale was 0.82, all other correlations were smaller than 0.54 (most of them substantially so). Critically, the CMQ fared very well – and much better than any other of the individual difference measures – in predicting beliefs in specific conspiracy theories even when controlling for a large number of alternative predictors. Only a minority of specific conspiracy theories could not be predicted well. Again, this may hint at the role of situational factors in determining the degree to which people (at the time of data collection) believed in these specific theories. This underlines the need to integrate research on situational factors determining endorsement of specific conspiracy beliefs with stable individual differences in conspiracy mentality.

We argue that our findings build a convincing case for the suitability and utility of the CMQ as a measure of conspiracy beliefs. However, we recognize that the present studies have their limitations. First, our samples were not representative of the general population of the different countries, and any generalization should therefore proceed with caution. However, one should note that we made an effort to recruit diverse participant samples to avoid relying on highly specific subgroups of the population (Henrich et al., 2010). In particular, samples differed in terms of participants’ professions (e.g., students in Studies 2 and 4 and employees in Study 3) as well as in cultural background (Western Europe, Middle East, and North America). Further, Study 1a is – with over 7,500 participants – one of the largest available data collections on a broad range of items assessing both generic and specific beliefs in conspiracy theories in a cross-cultural context. Similarity in recruitment methods across cultural groups resulted in comparable group compositions in terms of key socio-demographic characteristics, thus allowing for at least tentative between-country comparisons concerning the absolute level of conspiracy mentality. We are aware that the difference in sample size across the four studies (ranging from *N* = 76 to *N* = 7,766) may appear inconsistent. However, on close inspection the results reveal high levels of consistency of findings across the different studies. For example: (1) despite the differences in sample size, age, and professions between Studies 2 and 3, the CMQ scores are highly similar in terms of means and standard deviations. Further, (2) the correlation between the Paranoid Ideation Scale and the CMQ are of similar strength across the two studies (*r* = 0.45 and 0.50). (3) The findings concerning the CMQs predictive validity are highly consistent throughout all four studies. The average correlation of the CMQ with all specific conspiracy theory items always ranged between 0.50 and 0.58. More importantly – as stated above – the CMQ consistently turned out to be the strongest predictor for the belief in specific conspiracy theories across all studies, despite controlling for a broad range of other potential predictors. Second, we are aware of the fact that our scale is not completely free from content-related aspects. For example, the item “I think government agencies closely monitor all citizens” does provide more specific information than the item “I think that events which superficially seem to lack a connection are often the result of secret activities.” This difference may be responsible for the fact that the former item showed a comparatively low factor loading in the Turkish language version. Although we acknowledge that this potential problem must be carefully monitored when extending the use of the scale to further, possibly non-democratic, countries, the level of content provided still allowed for measurement equivalence across the language versions. Lastly, an instrument with items not mentioning any actors or possible topics of conspiracies runs the danger of not adequately capturing the construct. Thus, in our view, the distinction between scales measuring generic and specific conspiracy beliefs is not as clear cut as one may initially suspect. In particular, “generic” scales will usually have to provide some level of specific content to render the assessment of a conspiracy mentality meaningful. Our scale, we suggest, qualifies as a comparatively generic measure able to efficiently assess the general tendency to believe in conspiracy theories in a cross-cultural context.

## Coda

There is much reason to think that there is no return to what Moscovici (1987) describes as the amateur stage of conspiracy beliefs. To the contrary, an increasingly fast-paced political process characterized by frantic political and economic competition is bound to generate a number of competing theories proclaiming to explain certain societal phenomena and events. The internet is a powerful communication tool that even allows theories to proliferate that in former times may not have passed the filter of mainstream media; and conspiracy theories seem ubiquitous in many cultural contexts. Whether presidential candidates are blamed for covering their real birth place or whether outside powers are blamed for civil unrest in autocratic countries like Syria – conspiracy beliefs permeate political and societal processes. Being able to reliably assess the general tendency to endorse such theories with the CMQ should help to guide the next steps in the exciting endeavor to better understand the psychological role of such beliefs in social change and individual life courses.

## Conflict of Interest Statement

The authors declare that the research was conducted in the absence of any commercial or financial relationships that could be construed as a potential conflict of interest.

## Appendix

### Conspiracy belief questionnaire

Note: Response formats for each item in the respective language can be found beneath item wordings.

#### English

*I think that*…

**Table:**

1	…many very important things happen in the world, which the public is never informed about.
2	… politicians usually do not tell us the true motives for their decisions.
3	… government agencies closely monitor all citizens.
4	… events which superficially seem to lack a connection are often the result of secret activities.
5	… there are secret organizations that greatly influence political decisions.

**Table:**

0%	10%	20%	30%	40%	50%	60%	70%	80%	90%	100%
certainly not	extremely unlikely	very unlikely	unlikely	somewhat unlikely	undecided	somewhat likely	likely	very likely	extremely likely	certain

#### German

*Ich denke*…

**Table:**

1	… es geschehen viele sehr wichtige Dinge in der Welt, über die die Öffentlichkeit nie informiert wird.
2	… Politiker geben uns normalerweise keine Auskunft über die wahren Motive ihrer Entscheidungen.
3	… Regierungsbehörden überwachen alle Bürger genau.
4	… Ereignisse, die auf den ersten Blick nicht miteinander in Verbindung zu stehen scheinen, sind oft das Ergebnis geheimer Aktivitäten.
5	… es gibt geheime Organisationen, die großen Einfluss auf politische Entscheidungen haben.

**Table:**

0%	10%	20%	30%	40%	50%	60%	70%	80%	90%	100%
sicher nicht	Äußerst unwahrscheinlich	sehr unwahrscheinlich	unwahrscheinlich	eher unwahrscheinlich	unentschieden	eher wahrscheinlich	wahrscheinlich	sehr wahrscheinlich	Äußerst wahrscheinlich	sicher

#### Turkish

*Bana göre*…

**Table:**

1	… dünyada halka hiç bir sekilde haber verilmeden gerçeklesen çok fazla sayida önemli olaylar olmaktadir.
2	… siyasetçiler, genellikle, kararlarinin altinda yatan gerçek nedenleri bizlere açiklamazlar.
3	… hükümet ajanlari bütün vatandaslari yakindan izlemektedir.
4	… yüzeysel olarak ele alindiginda baglantisiz gibi görünen olaylar aslinda çogunlukla gizli aktivitelerin sonucudur.
5	… siyasi kararlari büyük ölçüde etkileyen gizli kuruluslar vardir.

**Table:**

%0	%10	%20	%30	%40	%50	%60	%70	%80	%90	%100
kesinlikle hayir	kesinlikle olasi degil	hiç olasi degil	olasi degil	pek olasi degil	kararsizim	biraz olasi	olasi	oldukça olasi	yüksek derecede olasi	kesinlikle evet
